# 3-(Pyridin-3-yl)propionic acid

**DOI:** 10.1107/S1600536810052384

**Published:** 2010-12-24

**Authors:** Andreas Lemmerer

**Affiliations:** a Molecular Sciences Institute, School of Chemistry, University of the Witwatersrand, Private Bag, PO WITS, 2050 Johannesburg, South Africa

## Abstract

In the crystal of the title compound, C_8_H_9_NO_2_, mol­ecules assemble to form *C*(8) chains along the *b* axis by N—H⋯O hydrogen bonds, supported by weaker C—H⋯O hydrogen bonded-inter­actions between adjacent chains.

## Related literature

For use of the title compound in coordination polymers, see: Wang *et al.* (2006 ▶). For graph-set nomenclature of hydrogen bonds, see: Bernstein *et al.* (1995 ▶).
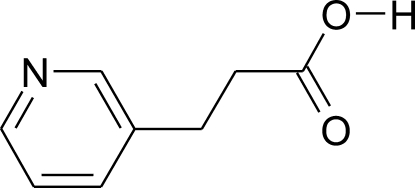


## Experimental

### 

#### Crystal data

C_8_H_9_NO_2_
*M* *_r_* = 151.16
Monoclinic, 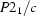

*a* = 6.7157 (4) Å
*b* = 14.6544 (13) Å
*c* = 7.2993 (6) Å
β = 92.566 (5)°
*V* = 717.64 (10) Å^3^
*Z* = 4
Mo *K*α radiation
μ = 0.10 mm^−1^
*T* = 173 K
0.42 × 0.27 × 0.04 mm

#### Data collection

Nonius KappaCCD area-detector diffractometer
Absorption correction: integration (*XPREP*; Bruker, 1999 ▶) *T*_min_ = 0.967, *T*_max_ = 0.995
10553 measured reflections
1729 independent reflections
1341 reflections with *I* > 2σ(*I*)
*R*_int_ = 0.051

#### Refinement

*R*[*F* ^2^ > 2σ(*F* ^2^)] = 0.040
*wR*(*F* ^2^) = 0.115
*S* = 1.02
1729 reflections
101 parameters
H-atom parameters constrained
Δρ_max_ = 0.26 e Å^−3^
Δρ_min_ = −0.26 e Å^−3^

### 

Data collection: *COLLECT* (Nonius, 1998 ▶); cell refinement: *DENZO* (Otwinowski & Minor, 1997 ▶) and *COLLECT*; data reduction: *DENZO* and *COLLECT*; program(s) used to solve structure: *SHELXS97* (Sheldrick, 2008 ▶); program(s) used to refine structure: *SHELXL97* (Sheldrick, 2008 ▶); molecular graphics: *ORTEP-3 for Windows* (Farrugia, 1997 ▶) and *DIAMOND* (Brandenburg, 1999 ▶); software used to prepare material for publication: *WinGX* (Farrugia, 1999 ▶) and *PLATON* (Spek, 2009 ▶).

## Supplementary Material

Crystal structure: contains datablocks global, I. DOI: 10.1107/S1600536810052384/fj2372sup1.cif

Structure factors: contains datablocks I. DOI: 10.1107/S1600536810052384/fj2372Isup2.hkl

Additional supplementary materials:  crystallographic information; 3D view; checkCIF report

## Figures and Tables

**Table 1:** Hydrogen-bond geometry (Å, °)

*D*—H⋯*A*	*D*—H	H⋯*A*	*D*⋯*A*	*D*—H⋯*A*
O1—H1⋯N1^i^	0.84	1.75	2.5868 (13)	172
C3—H3⋯O1^ii^	0.95	2.47	3.3468 (15)	154
C5—H5⋯O2^iii^	0.95	2.63	3.3328 (15)	131

Symmetry codes: (i) 
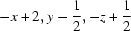
; (ii) 
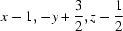
; (iii) 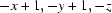.

**Fig. 1.**
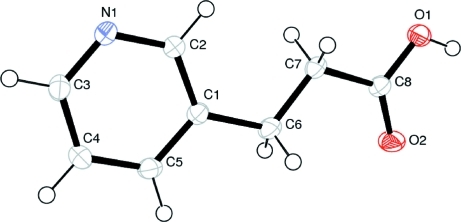
View of (I) (50% probability displacement ellipsoids)

**Fig. 2.**
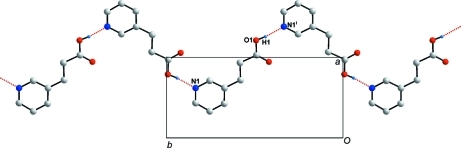
Hydrogen bonding C(8) chain of (I) using N—H···O hydrogen bonds (red dashed lines), generated by the twofold screw axis. Symmetry code: (i) -x + 2, y - 1/2, -z + 1/2.

## supplementary crystallographic information

### Comment

The molecule 3-pyridinepropionic acid (I) has been used as a ligand to make coordination polymers using Ag, Cu and Zn (Wang *et al.*, 2006). The crystal structure of the molecule itself has not been reported. The solid state packing involves forming chains of molecules, generated by hydrogen bonding interactions from the carboxylic acid H to the lone pair of the pyridine N atom. The chain, described using graph set notation (Bernstein *et al.*, 1995) as C(8), is generated by the twofold screw axis inherent in the spacegroup *P*2_1_/*c*, and runs along the crystallographic *b* axis (See Fig 2). Adjacent chains are stabilized by two C—H···O hydrogen bonds (See Table 1).

### Experimental

Crystals where grown by slow evaporation at ambient conditions of a methanol solution of 3-pyridinepropionic acid.

### Refinement

The C-bound H atoms were geometrically placed (C—H bond lengths of 0.95 (aromatic CH) and 0.99 (CH_2_) Å) and refined as riding with *U*_iso_(H) = 1.2*U*_eq_(C). The O-bound H atom were geometrically placed and refined as riding with *U*_iso_(H) = 1.5*U*_eq_(O).

### Crystal data

**Table:**

C_8_H_9_NO_2_	*F*(000) = 320
*M**_r_* = 151.16	*D*_x_ = 1.399 Mg m^−^^3^
Monoclinic, *P*2_1_/*c*	Mo *K*α radiation, λ = 0.71073 Å
Hall symbol: -P 2ybc	Cell parameters from 3866 reflections
*a* = 6.7157 (4) Å	θ = 0.4–30.0°
*b* = 14.6544 (13) Å	µ = 0.10 mm^−^^1^
*c* = 7.2993 (6) Å	*T* = 173 K
β = 92.566 (5)°	Plate, colourless
*V* = 717.64 (10) Å^3^	0.42 × 0.27 × 0.04 mm
*Z* = 4	

### Data collection

**Table:**

Nonius KappaCCD area-detector diffractometer	1341 reflections with *I* > 2σ(*I*)
2.0° π and ω scans	*R*_int_ = 0.051
Absorption correction: integration (*XPREP*; Bruker, 1999)	θ_max_ = 28°, θ_min_ = 3.0°
*T*_min_ = 0.967, *T*_max_ = 0.995	*h* = −8→7
10553 measured reflections	*k* = −19→19
1729 independent reflections	*l* = −9→9

### Refinement

**Table:**

Refinement on *F*^2^	0 restraints
Least-squares matrix: full	H-atom parameters constrained
*R*[*F*^2^ > 2σ(*F*^2^)] = 0.040	*w* = 1/[σ^2^(*F*_o_^2^) + (0.0616*P*)^2^ + 0.1772*P*] where *P* = (*F*_o_^2^ + 2*F*_c_^2^)/3
*wR*(*F*^2^) = 0.115	(Δ/σ)_max_ < 0.001
*S* = 1.02	Δρ_max_ = 0.26 e Å^−^^3^
1729 reflections	Δρ_min_ = −0.26 e Å^−^^3^
101 parameters	

### Special details

**Table:**

Experimental. Numerical integration absorption corrections based on indexed crystal faces were applied using the *XPREP* routine (Bruker, 1999)
Geometry. All e.s.d.'s (except the e.s.d. in the dihedral angle between two l.s. planes) are estimated using the full covariance matrix. The cell e.s.d.'s are taken into account individually in the estimation of e.s.d.'s in distances, angles and torsion angles; correlations between e.s.d.'s in cell parameters are only used when they are defined by crystal symmetry. An approximate (isotropic) treatment of cell e.s.d.'s is used for estimating e.s.d.'s involving l.s. planes.

### Fractional atomic coordinates and isotropic or equivalent isotropic displacement parameters (Å^2^)

**Table:**

	*x*	*y*	*z*	*U*_iso_*/*U*_eq_	
C1	0.61989 (17)	0.67074 (7)	0.12796 (15)	0.0187 (3)	
C2	0.71028 (18)	0.75445 (8)	0.16533 (16)	0.0198 (3)	
H2	0.8419	0.755	0.2186	0.024*	
C3	0.43377 (18)	0.83489 (8)	0.05545 (16)	0.0229 (3)	
H3	0.3693	0.8916	0.0312	0.028*	
C4	0.33224 (19)	0.75458 (8)	0.01309 (18)	0.0242 (3)	
H4	0.2003	0.7561	−0.0395	0.029*	
C5	0.42692 (18)	0.67253 (8)	0.04903 (16)	0.0221 (3)	
H5	0.3602	0.6169	0.0198	0.026*	
C6	0.71897 (18)	0.57984 (8)	0.16581 (17)	0.0236 (3)	
H6A	0.7149	0.5443	0.0503	0.028*	
H6B	0.6391	0.5461	0.2543	0.028*	
C7	0.93261 (17)	0.58303 (8)	0.24056 (16)	0.0215 (3)	
H7A	0.9366	0.6121	0.3631	0.026*	
H7B	1.0116	0.6214	0.159	0.026*	
C8	1.02756 (18)	0.48938 (7)	0.25664 (16)	0.0206 (3)	
N1	0.61911 (15)	0.83489 (6)	0.12917 (14)	0.0212 (3)	
O1	1.21073 (13)	0.49226 (6)	0.32765 (13)	0.0294 (3)	
H1	1.2566	0.439	0.3362	0.044*	
O2	0.94438 (13)	0.41935 (6)	0.20706 (13)	0.0315 (3)	

### Atomic displacement parameters (Å^2^)

**Table:**

	*U*^11^	*U*^22^	*U*^33^	*U*^12^	*U*^13^	*U*^23^
C1	0.0190 (6)	0.0172 (6)	0.0201 (6)	0.0000 (4)	0.0022 (4)	0.0003 (4)
C2	0.0191 (6)	0.0182 (6)	0.0220 (6)	−0.0004 (4)	−0.0009 (4)	0.0008 (4)
C3	0.0229 (6)	0.0203 (6)	0.0255 (6)	0.0032 (5)	0.0000 (5)	0.0035 (4)
C4	0.0187 (6)	0.0262 (6)	0.0273 (6)	−0.0001 (5)	−0.0029 (5)	0.0026 (5)
C5	0.0214 (6)	0.0195 (6)	0.0254 (6)	−0.0038 (4)	0.0007 (5)	−0.0011 (4)
C6	0.0223 (6)	0.0152 (5)	0.0334 (7)	−0.0005 (4)	0.0004 (5)	−0.0006 (5)
C7	0.0224 (6)	0.0145 (5)	0.0276 (6)	0.0003 (4)	0.0002 (5)	0.0002 (4)
C8	0.0218 (6)	0.0173 (6)	0.0228 (6)	−0.0006 (4)	0.0017 (5)	−0.0001 (4)
N1	0.0231 (5)	0.0166 (5)	0.0238 (5)	−0.0002 (4)	−0.0004 (4)	0.0011 (4)
O1	0.0255 (5)	0.0153 (4)	0.0462 (6)	0.0024 (3)	−0.0115 (4)	−0.0022 (4)
O2	0.0256 (5)	0.0172 (4)	0.0510 (6)	−0.0014 (3)	−0.0074 (4)	−0.0048 (4)

### Geometric parameters (Å, °)

**Table:**

C1—C2	1.3903 (16)	C5—H5	0.95
C1—C5	1.3947 (17)	C6—C7	1.5127 (16)
C1—C6	1.5090 (16)	C6—H6A	0.99
C2—N1	1.3489 (15)	C6—H6B	0.99
C2—H2	0.95	C7—C8	1.5156 (15)
C3—N1	1.3338 (16)	C7—H7A	0.99
C3—C4	1.3881 (17)	C7—H7B	0.99
C3—H3	0.95	C8—O2	1.2157 (14)
C4—C5	1.3798 (17)	C8—O1	1.3140 (15)
C4—H4	0.95	O1—H1	0.84
			
C2—C1—C5	117.00 (10)	C1—C6—H6A	108.2
C2—C1—C6	123.91 (10)	C7—C6—H6A	108.2
C5—C1—C6	119.09 (10)	C1—C6—H6B	108.2
N1—C2—C1	122.84 (11)	C7—C6—H6B	108.2
N1—C2—H2	118.6	H6A—C6—H6B	107.4
C1—C2—H2	118.6	C6—C7—C8	112.89 (9)
N1—C3—C4	122.01 (11)	C6—C7—H7A	109
N1—C3—H3	119	C8—C7—H7A	109
C4—C3—H3	119	C6—C7—H7B	109
C5—C4—C3	118.62 (11)	C8—C7—H7B	109
C5—C4—H4	120.7	H7A—C7—H7B	107.8
C3—C4—H4	120.7	O2—C8—O1	123.65 (10)
C4—C5—C1	120.44 (11)	O2—C8—C7	123.72 (11)
C4—C5—H5	119.8	O1—C8—C7	112.62 (9)
C1—C5—H5	119.8	C3—N1—C2	119.09 (10)
C1—C6—C7	116.24 (10)	C8—O1—H1	109.5
			
C5—C1—C2—N1	0.27 (18)	C5—C1—C6—C7	176.33 (10)
C6—C1—C2—N1	179.56 (11)	C1—C6—C7—C8	−174.20 (10)
N1—C3—C4—C5	0.09 (19)	C6—C7—C8—O2	3.16 (17)
C3—C4—C5—C1	0.63 (18)	C6—C7—C8—O1	−177.75 (10)
C2—C1—C5—C4	−0.79 (18)	C4—C3—N1—C2	−0.62 (18)
C6—C1—C5—C4	179.88 (10)	C1—C2—N1—C3	0.43 (18)
C2—C1—C6—C7	−2.95 (17)		

### Hydrogen-bond geometry (Å, °)

**Table:**

*D*—H···*A*	*D*—H	H···*A*	*D*···*A*	*D*—H···*A*
O1—H1···N1^i^	0.84	1.75	2.5868 (13)	172
C3—H3···O1^ii^	0.95	2.47	3.3468 (15)	154
C5—H5···O2^iii^	0.95	2.63	3.3328 (15)	131

Symmetry codes: (i) −*x*+2, *y*−1/2, −*z*+1/2; (ii) *x*−1, −*y*+3/2, *z*−1/2; (iii) −*x*+1, −*y*+1, −*z*.
